# Exploring the Spectrum of Kidney Ciliopathies

**DOI:** 10.3390/diagnostics10121099

**Published:** 2020-12-16

**Authors:** Matteo Santoni, Francesco Piva, Alessia Cimadamore, Matteo Giulietti, Nicola Battelli, Rodolfo Montironi, Laura Cosmai, Camillo Porta

**Affiliations:** 1 Oncology Unit, Macerata Hospital, 62100 Macerata, Italy; nicola.battelli@sanita.marche.it; 2 Department of Specialistic Clinical and Odontostomatological Sciences, Polytechnic University of Marche, 60126 Ancona, Italy; f.piva@staff.univpm.it (F.P.); m.giulietti@staff.univpm.it (M.G.); 3 Section of Pathological Anatomy, School of Medicine, United Hospitals, Polytechnic University of the Marche Region, 60126 Ancona, Italy; r.montironi@staff.univpm.it; 4 Division of Nephrology and Dialysis, ASST Fatebenefratelli-Sacco, Fatebenefratelli Hospital, 20121 Milan, Italy; lacos@iol.it; 5 Chair of Oncology, Department of Biomedical Sciences and Human Oncology, University of Bari ‘A. Moro’, 70121 Bari, Italy; camillo.porta@gmail.com; 6 Division of Medical Oncology, A.O.U. ConsorzialePoliclinico di Bari, 70124 Bari, Italy

**Keywords:** ciliopathies, fibrocystin, polycystin-1, polycystin-2, polycystic kidney disease, kidney, renal cell carcinoma

## Abstract

Ciliopathies are a group of multi-organ diseases caused by the disruption of the primary cilium. This event leads to a variety of kidney disorders, including nephronophthisis, renal cystic dysplasia, and renal cell carcinoma (RCC). Primary cilium contributes to the regulation of the cell cycle and protein homeostasis, that is, the balance between protein synthesis and degradation by acting on the ubiquitin-proteasome system, autophagy, and mTOR signaling. Many proteins are involved in renal ciliopathies. In particular, fibrocystin (PKHD1) is involved in autosomal recessive polycystic kidney disease (ARPKD), while polycystin-1 (PKD1) and polycystin-2 (PKD2) are implicated in autosomal dominant polycystic kidney disease (ADPKD). Moreover, primary cilia are associated with essential signaling pathways, such as Hedgehog, Wnt, and Platelet-Derived Growth Factor (PDGF). In this review, we focused on the ciliopathies associated with kidney diseases, exploring genes and signaling pathways associated with primary cilium and the potential role of cilia as therapeutic targets in renal disorders.

## 1. Introduction

Flagella and cilia in eukaryotic cells have been historically considered as organelles involved in cell motility [1]. The structure of the motile cilia, which are shorter and more numerous than flagella (>100 copies for each cell), is composed by a basal body and an axoneme with a 9 + 0 or 9 + 2 arrangement of microtubules. The function of cilia results in cell motility through water (typical of a single cell organism) or in moving water and its content across the cell surface (as for the motility of mucus within the human respiratory tract). Beyond motile cilia, Alexander Kowalevsky discovered in 1867 [1] the presence of a single nonmotile cilia, the primary cilium, which lacks the central pair of microtubules [2] and is present in almost all eukaryotic cells. Primary cilia can transduce extracellular signals acting as mechano- or photoreceptors, but they can also be involved in detecting, for example, osmolarity, chemicals, and temperature [3]. Moreover, primary cilia are associated with essential signaling pathways, such as Hedgehog, Wnt, and Platelet-Derived Growth Factor (PDGF) [4,5,6].

In the last decade, the advances in proteomics and genomics have led to the creation of a “ciliome” [7,8,9], a powerful collection of over 3000 genes encoding proteins involved in cilia assembly and functions [10]. For example, this list includes RFX-type transcription factor DAF-19, which regulates proteins implicated in intraflagellar transport [11,12], MKS1 (Meckel Syndrome Type 1), BBS3 (Bardet-biedl syndrome 3), and BBS5 (Bardet-biedl syndrome 5) [13,14,15,16]. Beyond these, a series of proteins that indirectly interact with ciliary components has been identified [17,18].

Ciliopathies are rare conditions that have emerged as a new challenge for worldwide researchers [19]. Alterations of ciliary motility can lead to a wide spectrum of specific diseases in humans, including, for example, sterility for defective sperm cilia and a condition known as “situs inversus”, in which internal organs are inverted due to defective embryonic cilia. 

For the purposes of this review, we focused exclusively on the ciliopathies associated with kidney disorders, from renal cystic dysplasia to cancer, exploring proteins and signaling pathways associated with primary cilium and the potential role of cilia as therapeutic targets in renal diseases. 

## 2. Genes and Signaling Pathways in the Primary Cilia

The cilium is formed by proteins and other molecules transported along the axoneme by the intraflagellar transport (IFT) particles, since protein translation does not occur in the cilia. Dynein 2/1b performs the intraflagellar transport from the base to the tip of cilium, whereas kinesin 2 carries the cargo in the opposite direction. The intraflagellar transport is important to ensure the normal functioning of the cilia. Indeed, the disruption of IFT88 (intraflagellar transport protein 88 homolog) results in polycystic kidney disease in mice [20]. Many other genes are involved in ciliopathies, in particular, fibrocystin (*PKHD1*) is implicated in autosomal recessive polycystic kidney disease (ARPKD), polycystin-1 (*PKD1*) and polycystin-2 (*PKD2*) are associated to autosomal dominant polycystic kidney disease (ADPKD), and nephrocystins are correlated with nephronophthisis. However, a list of genes involved in cilia-associated human cystic kidney disease can be found in the review by Dell et al. [21]. Among the genes not included in this list are cyclin-dependent-like kinase 5 (CDK5), a kinase regulated by p35 (cyclin-dependent kinase 5 activator 1) and p39 (cyclin-dependent kinase 5 activator 2). CDK5 hyper activation can promote renal cystogenesis, and its inhibition reduces ciliary length, restores cell differentiation, and attenuates disease progression [22].

The presence of cilia is regulated during cell cycle progression. Indeed, cilia are resorbed prior to entry into mitosis, allowing the centrioles’ detachment from the basal body. Therefore, it seems that primary cilia can prevent uncontrolled cell growth, as proved also by the role of IFT88 (intraflagellar transport protein 88 homolog) and NDE1 (nuclear distribution protein nudE homolog 1) proteins in controlling cell division. In particular, IFT88 is tightly associated with the centrosome, and influences the G1-S cell cycle progression [23]. On the other hand, centrosomal phosphoprotein NDE1 is a negative regulator of ciliary length that acts by modulating the IFT process. The NDE1 expression level is reduced when the cell enters G1 and is high in mitosis, thus ciliary length is regulated in a cell cycle-dependent manner. At the molecular level, CDK5 seems to regulate NDE1 degradation through FBW7 E3 ubiquitin ligase [24]. Another element that controls the cell cycle is NDEL1 (nuclear distribution protein nudE-like 1), a scaffold protein that regulates microtubule dynamics and microtubule-based transport. Its expression reduces cilia length in proliferating cells, which promotes cell cycle progression [25].

It is notable that, in patients with clear cell RCC with mutations in the *VHL* (von Hippel-Lindau disease tumor suppressor) gene, primary cilia have been lost, and the re-expression of VHL proteins restored cilia expression [26]. Although cell proliferation affects the presence of cilia, renal cancer cells do not contain primary cilia independently of levels of Ki67 cell proliferation marker [27]. In other words, the loss of cilia would be independent from the rate of cell proliferation.

Primary cilium also contributes to the regulation of protein homeostasis, that is, the balance between protein synthesis and degradation, by acting on the ubiquitin-proteasome system, autophagy, and mTOR signaling. In fact, several proteasome proteins are present in cilia, and contribute to their disassembly during the cell cycle. By autophagy, proteins and organelles are degraded in order to supply amino acids to sustain ciliogenesis, but autophagy can also limit ciliogenesis by eliminating components of ciliary transport; mTOR can promote ciliogenesis by limiting autophagy and increasing protein and lipid synthesis, glycolysis, and oxidative metabolism [28].

**Figure 1:**
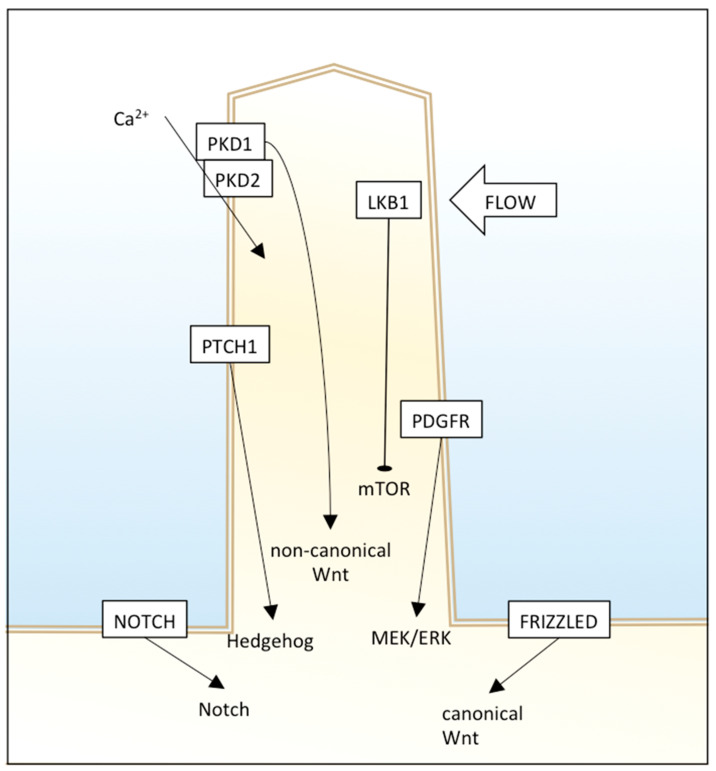
The primary cilium is a “central processing unit” of the cell that integrates different extracellular signals to regulate cell functioning. Different signaling pathways are shown.

The primary cilium has different signaling receptors on the ciliary membrane, including those for Hedgehog (Hh), Notch, mTOR, platelet-derived growth factor receptor (PDGFR), and canonical and non-canonical Wnt pathways (Figure 1).

Hedgehog signaling is involved in vertebrate embryonic development and stem cell maintenance, and its dysregulation was found in many human tumors. Primary cilia have an essential role in Hh signaling; in fact, some genes are required both for Hh signaling and primary cilia formation. PTCH1 (protein patched homolog 1), the receptor for Hh ligands, resides in the cilium membrane and represses and averts SMO (smoothened homolog). In the unstimulated state, GLI transcription factors are suppressed by SUFU (suppressor of fused homolog). Upon binding of an Hh ligand to PTCH1, SMO is not repressed anymore, and enters the cilium instead of PTCH1. SMO represses SUFU, and this causes GLIs release and activation. The movement of these Hh proteins along cilium is facilitated by IFT proteins and motor proteins, therefore, loss of IFT proteins leads to Hh alteration [29]. However, the control of this pathway by cilia is complex and context-dependent, as nonfunctional or lack of cilia can result in inhibition or enhancement of Hh signaling. For example, in two Hh pathway-dependent mouse basal cell carcinoma models, cilia deletion was able to inhibit tumor growth induced by an activated form of SMO, but also promote tumor growth induced by activated GLI2 [30]. This suggests that primary cilia could promote or inhibit carcinogenesis through Hh depending on the nature of the oncogenic initiating event.

Wnt signaling is involved in cell migration, planar cell polarity, and organogenesis, but its alterations are related to different cancer types. Many published papers suggest that primary cilia have an important role in attenuation of a canonical and non-canonical (β-catenin independent) Wnt signaling pathway. Defects in cilia cause over-activation of Wnt, leading to cystic kidney disease in a mouse model [31] and Joubert syndrome in humans [32]. Moreover, Wnt ligands can bind to PKD1 and, through the PKD2 channel, induce Ca(2+) influx [33]. An interesting work showed that carcinogens ochratoxin A (OTA) and potassium bromate (KBrO3) induced loss of the primary cilium in a human proximal tubular epithelial cell line and the dysregulation of different pathways, including Wnt signaling and cytoskeletal remodeling [34]. Since beta-catenin is sequestered in the primary cilium, an undamaged primary cilium that retains the effector efficiently acts as a negative regulator of Wnt signaling, and deciliation could enhance Wnt, leading to the progression of malignant tumors.

The PDGF pathway is involved in cell growth, proliferation, migration, and embryonic development, and its dysregulation causes different diseases, including cancer.

PDGFRA is present in primary cilia membranes, and cells lacking normal cilia cannot activate MEK1/2 and ERK1/2 [35,36].

Notch signaling is involved in cell fate determination. Some Notch components reside in cilia, and loss of the latter alters Notch functioning, therefore, differentiation of basal cells to spinous cells. In particular, Notch3 localizes in the ciliary membrane and interacts with Presenilin-2, an enzyme localized to the ciliary basal body and responsible for Notch cleavage and activation. Notch signaling also regulates cilium length and trafficking of Hh mediators into primary cilium [35].

## 3. Renal Ciliopathies

### 3.1. Nephronophthisis

Nephronophthisis (NPHP) is inherited in an autosomal recessive mode and represents, although rare, the most frequent cause of end-stage renal disease (ESRD) in children and young adults [37], with a prevalence of 1/100,000 individuals. Although usually characterized by aspecific symptoms (i.e., urine concentration, polydipsia, and polyuria), in 10–20% of cases, NPHP presents additional features of ciliopathy syndrome, such as retinal defects, liver fibrosis, skeletal abnormalities, and brain developmental disorders [38].

Thus, diagnosis usually requires genetic tests, renal ultrasounds, and biopsies. At ultrasound, kidneys usually present normal size, with an increase in echogenicity sometimes associated with renal cysts. At biopsy, NPHP shows tubular basement lesions, which are thickened and multilayered, interstitial fibrosis, and inflammation [37].

Traditional classification of NPHP is based on the age of onset of ESRD: infantile, juvenile, or adolescent/adult. The most common form, juvenile NPHP, is also called classic NPHP [39].

**Figure 2:**
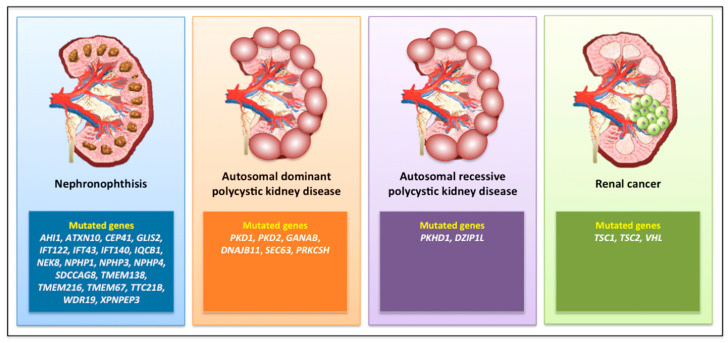
A selection of gene mutations associated with the development of renal ciliopathies.

NPHP has been correlated with mutations in at least 25 different genes (Figure 2) involved in renal development and homeostasis [40].

The majority of these genes encode proteins interconnected in a nephrocystin protein complex that resides at the transition zone. Approximately 70% of NPHP are still genetically unclear, while mutations in the *NPHP1* gene are responsible for 20% of all cases. This gene has been mapped to chromosome 2q13, firstly identified in 1997 [41], and codes for Nephrocystin-1, which is an adaptor protein mainly localized in primary cilia and the apical surface of renal epithelial cells, together with other adhesion and signaling transducer molecules. *NPHP1* alterations are associated not only with NPHP, but also with neurological disorders, including Joubert syndrome, described by cerebellar vermis hypoplasia and brainstem abnormalities [42]; Senior–Løken syndrome, consisting of retinal degeneration to blindness; Bardet–Biedl syndrome, with intellectual disability and obesity [43]; and skeletal ciliopathies characterized by bone shortening (i.e., Jeune asphyxiating thoracic dystrophy [44] and Sensenbrenner syndrome [45]).

Meckel–Gruber syndrome is a highly lethal perinatal autosomal recessive congenital anomaly syndrome mapped to six different loci in different chromosomes; it has an extreme genetic heterogeneity, highly variable phenotype (due to multi-organ involvement), and displays allelism with other related ciliopathies, presenting significant challenges for diagnosis [46].

### 3.2. Autosomal Dominant Polycystic Kidney Disease

Autosomal dominant polycystic kidney disease (ADPKD), the most common inherited cause of renal failure, is generally an adult-onset condition with a prevalence of 1/1000, characterized by gradually growing fluid-filled renal cysts originating from all areas of the kidneys, associated with hepatobiliary changes, hypertension, and/or other extra-renal abnormalities (i.e., left ventricular hypertrophy and intracranial arterial aneurysms) [47,48].

The most common causes are mutations in *PKD1* (80% of cases) or *PKD2* (15% of cases), encoding two components of a heterodimeric complex (polycystin 1, PC1, and PC2) co-localized in the primary cilium, where they transduce the extracellular fluid flow shear stress into a Ca2+ signal [49,50]. Beyond *PKD1* and *PKD2* (which present with high levels of allelic heterogeneity [51]), a series of mutated genes has been described in ADPKD, such as neutral α-glucosidase AB (*GANAB*) [52], *DNAJB11* [53], *SEC63*, and *PRKCSH* [54] (Figure 2).

Though fully penetrant, it has a considerable phenotypic variability, conditioned by factors such as gender, type of mutation, and concomitant acquired diseases, such as hypertension and chronic kidney disease [55].

Cysts arise from the epithelia of only 1 to 5% of all nephrons, and are localized mainly in the distal convoluted tubule and collecting duct. They are lined by a single layer of rapidly proliferating and less differentiated tubular cells. The inflammatory tissue surrounding these cysts is induced by the secretion of cytochines and chemokines from the epithelium of the cysts.

Clinical symptoms include hypertension, abdominal pain, haematuria, and urinary tract infections [48]. The radiologic diagnosis is based on ultrasonography, which should often be accompanied by CT or MRI in order to obtain more quantitative data. Indeed, in ADPKD, the kidneys are markedly enlarged with vascular remodeling and interstitial fibrosis, while benign adenomas are present in about 25% of patients [56]. Genetic diagnosis of ADPKD should be performed by next-generation sequencing (NGS), caused by the complexity of the *PKD1* gene structure.

### 3.3. Autosomal Recessive Polycystic Kidney Disease

Autosomal recessive polycystic kidney disease (ARPKD) is much rarer (1/10,000) than ADPKD, and is generally a perinatal or childhood disease; indeed, 50% of the subjects develop ESRD from this disease at age 10 years or less. ARPKD is due to mutations in the Polycystic Kidney and Hepatic Disease 1 (*PKHD1*) gene, which encodes fibrocystin, and is localized to the primary cilium in the kidneys, liver, and pancreas, predominantly in the basal body of cilia present in renal tubular cells and biliary epithelial cells [57,58] (Figure 2). Fibrocystin is a key regulator of cell proliferation, apoptosis, and polarization [59]. Mutations in the gene encoding the ciliary transition zone protein DAZ-interacting protein 1-like protein (DZIP1L) have also been reported in ARPKD without evidence of *PKHD1* mutations [60] (Figure 2).

The clinical symptoms of ARPKD include cysts localized in the collecting ducts, with large kidneys at ultrasound, and congenital hepatic fibrosis (CHF). The diagnosis of ARPKD is built on clinical imaging in serious neonatal or infantile cases, and should require NGS testing for a more efficient diagnostic approach.

### 3.4. Renal Cystic Dysplasia

Cystic renal dysplasia is a congenital condition characterized by the presence of cysts in the renal cortex, distended collecting ducts, and poorly developed medullary pyramids [37]. Fibrous tissue may also be present [61]. The diagnosis of congenital renal cystic dysplasia is usually performed by ultrasonography prenatally or during early childhood. Cystic renal dysplasia may be associated with several urologic abnormalities, including ureteropelvic and ureterovesicular junction obstruction, neurogenic bladder, ureterocele, and prune belly syndrome [37].

### 3.5. Rare Renal Phenotypes

Beyond the renal ciliopathies described above, a series of less common phenotypes have been described, including horseshoe kidneys [62] and lobulated kidneys [63]. Moreover, cilium dysfunction can increase the risk of urinary tract infections [64] and kidney stones [65].

### 3.6. Renal Cell Carcinoma

**Figure 3:**
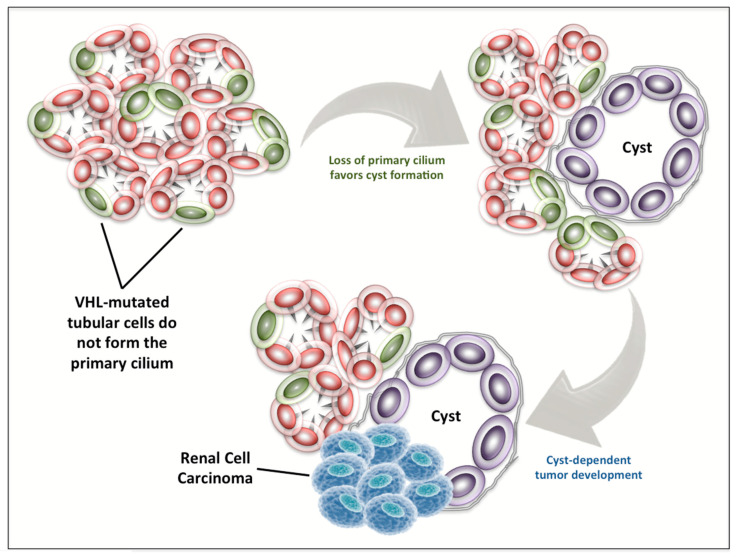
VHL loss in renal carcinogenesis. VHL-mutated tubular cells have been shown to not form the primary cilium. This event favors cyst formation and can lead to the development of renal cell carcinoma.

The connection between ciliogenesis, the formation of renal cysts, and the development of RCC is supported by a series of evidence published in the last 15 years [66,67,68,69,70,71,72,73]. Indeed, in 2004, Lolkema et al. [74] firstly showed that VHL protein can modulate tubulin turnover, thus influencing cellular migration, polarization, and cell–cell interactions. Two years later, it was reported that VHL can control ciliogenesis by orienting microtubule growth through the interaction with the Par3–Par6–atypical PKC complex [75]. In the same year, it became clear that VHL inactivation is associated with abrogation of the primary cilium in renal cysts of patients with VHL disease and in VHL-defective cell lines [76] (Figure 3), and this role of VHL for ciliogenesis is independent of hypoxia-induced factor (HIF)-α abundance, suggesting two distinct functions of VHL both necessary in RCC carcinogenesis [77].

In 2008, Lolkema et al. evidenced the essential activity of VHL for the initiation of ciliogenesis and in the maintenance of cilia in kidney cells [78]. They observed that reduced cellular levels of VHL led to a drop in ciliated cell frequency in mouse kidney cell lines. Moreover, only cells expressing VHL were able to respond to flow by rapidly increasing the intracellular Ca2+ concentrations, thus underlining the fundamental role of VHL in the mechanotransduction activity of primary cilia [78].

## 4. Conclusions

The advances in our knowledge about the genetic mechanisms underlying renal ciliopathies have improved the diagnosis and prognosis of these patients. The availability of NGS, together with bioinformatic analysis, will further expand our insights on ciliopathies, thus accelerating the route to the development of targeted approaches aimed at delaying or preventing the effects of this group of diseases. The fundamental role of ciliogenesis in RCC carcinogenesis opens a window to the possibility of developing strategies for the early phases of this disease. Nevertheless, the route to personalized approaches in patients with renal ciliopathies will probably require years of research and an even more straight collaboration between nephrologists, geneticists, and oncologists.
